# One-Dimensional Flow of Bacteria on an Electrode Rail by Dielectrophoresis: Toward Single-Cell-Based Analysis

**DOI:** 10.3390/mi12020123

**Published:** 2021-01-24

**Authors:** Yukihiro Yamaguchi, Takatoki Yamamoto

**Affiliations:** Department of Mechanical Engineering, School of Engineering, Tokyo Institute of Technology, 2-12-1 Ookayama, Meguro-ku, Tokyo 152-8550, Japan; yamaguchi.y.ay@m.titech.ac.jp

**Keywords:** dielectrophoresis, microfluidics, single cell, single bacterium, flow cytometer

## Abstract

Many applications in biotechnology and medicine, among other disciplines, require the rapid enumeration of bacteria, preferably using miniaturized portable devices. Microfluidic technology is expected to solve this miniaturization issue. In the enumeration of bacteria in microfluidic devices, the technique of aligning bacteria in a single line prior to counting is the key to an accurate count at single-bacterium resolution. Here, we describe the numerical and experimental evaluation of a device utilizing a dielectrophoretic force to array bacteria in a single line, allowing their facile numeration. The device comprises a channel to flow bacteria, two counter electrodes, and a capture electrode several microns or less in width for arranging bacteria in a single line. When the capture electrode is narrower than the diameter of a bacterium, the entrapment efficiency of the one-dimensional array is 80% or more within 2 s. Furthermore, since some cell-sorting applications require bacteria to move against the liquid flow, we demonstrated that bacteria can move in a single line in the off-axial direction tilted 30° from the flow direction. Our findings provide the basis for designing miniature, portable devices for evaluating bacteria with single-cell accuracy.

## 1. Introduction

Technologies for measuring the number of bacteria in a sample are important in biotechnology, medicine, the food industry, and hygiene management. For example, in the field of medical care, measuring the number of bacteria in the mucosa of a patient can be used to detect an abnormality in the body. In the food industry, the number of bacteria is monitored to control fermentation and to prevent food poisoning [1,2]. Such applications require rapid and precise bacterial measurement techniques, preferably using devices that are small and portable and thus suitable for on-site analysis such as environmental monitoring and point-of-care [3,4]. This need has led to the recent development and widespread use of small bacterial measurement devices using microfluidics technology and particularly miniaturized flow cytometers for separating and counting cell types at the single-cell level. Various methods have been developed based on optical detection [5,6], electrical impedance response [7,8], the Coulter counter method (nanopore measurement) [9], Raman spectroscopy [10,11], and a magnetic method involving binding magnetic beads to bacteria [12,13]. All these methods require the separation of bacteria into a single line which is fed to the measurement portion of the flow cytometer where the bacteria are processed individually. This is realized in a simple manner by limiting the channel diameter to that of a bacterium [14], but such channels tend to get clogged with bacteria. Conventionally, the flow diameter is limited to that of a bacterium using a sheath flow method, wrapping the sample flow with another flow stream [15,16]. This approach allows high-throughput counting but is difficult to miniaturize due to the complicated liquid delivery system. Stable sheath flow requires precise control of the flow rate and sample dilution by the sheath flow. Furthermore, problems such as damage to the cells caused by exposure to high pressure and shear forces caused by the sheath flow have been pointed out [17]. Sheathless methods have also been investigated, such as surface acoustic waves [18,19], the use of inertial force [20,21], and the electrostatic force of dielectrophoresis (DEP). However, the equipment for generating surface acoustic waves is expensive and non-disposable, and the equipment for using inertial force must be redesigned for each bacterial type and is not ideal for submicron particles such as bacteria. There are many reports on arranging mammalian cells in a single line using DEP [22,23,24] but few reports on arranging bacteria. In addition, most previous work used negative DEP [25]. Negative DEP acts toward the weak portion of an electric field, which has the advantage of causing less damage to the cells due to the electric field. However, this approach has the disadvantage that a steric and complex electrode structure is required to stably generate the force [26]. Furthermore, the conductivity of the solvent must be higher than that of the cell, causing wear of the electrode due to the electrode reaction and the high amount of heat generated, making it difficult to generate a strong dielectrophoretic force [27]. In contrast, a positive DEP acts toward the electrode edge where the electric field is strongest. However, the potential drawback of this approach is that it may damage cells due to the strong electric field and because the conductivity of the solvent must be lower than that of the cell [28].

At the same time, the low conductivity of the solution provides the advantage that a strong DEP can be applied because heat generation and the electrode reaction are suppressed more than in a negative DEP. The volume of a bacterial cell is less than 1/1000th that of mammalian cells, and thus, a positive dielectrophoretic force is preferable to effectively utilize the volumetric force of the dielectrophoretic force. However, there have been few reports on bacterial alignment by positive DEP and no reports of a technique in which bacterial cells are continuously arranged in a single line and fed individually downstream, although one example of generating a single stream of a cluster consisting of a small number of cells has been reported [29].

Here, we propose an electrode-rail structure to realize a technique to arrange bacterial cells in a single line using positive DEP. Our approach alleviates damage caused by the electric field by avoiding the electrode edges, where the greatest damage is caused by the electric field. We demonstrate the generation of a single line of bacteria on the electrode-rail by first analyzing the proposed device using the finite element method, then by fabricating the device, and by experimentally evaluating it based on the conditions obtained by the numerical analysis.

## 2. Materials and Methods

### 2.1. Design of the Device

**Figure 1:**
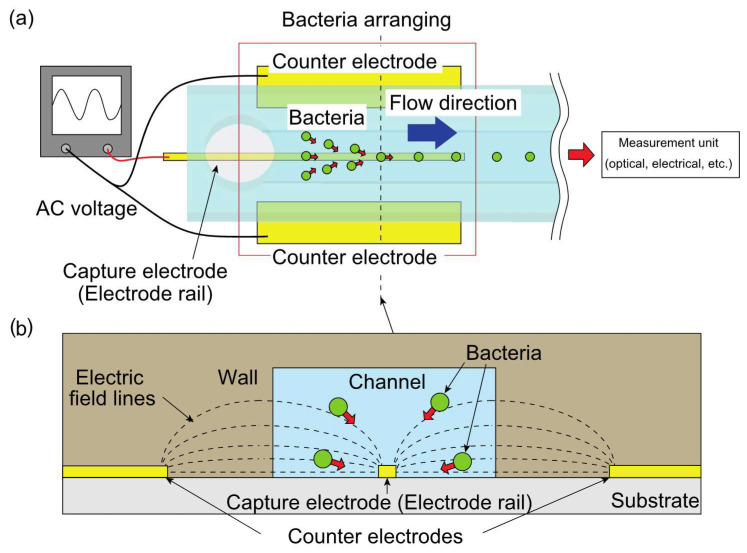
Schematic of the device used to form a line of bacterial cells: (**a**) top view of the device and connection of the AC power source to the electrodes and (**b**) cross-sectional view of the device. The dotted lines indicate the electric field lines which pass through the microchannel wall, creating a large electric field gradient to generate dielectrophoresis (DEP). Bacteria are attracted on the capture electrode (electrode rail).

A schematic diagram of the device is shown in Figure 1a. This device delivers the bacteria in a single line downstream to a flow cytometer and other bacterial single-cell analysis devices. The device comprises a flow channel to flow bacteria, two counter electrodes, and a capture electrode (electrode rail) several microns or less in width for arranging bacteria in a single row.

The electric field is generated using one capture electrode and two counter electrodes located outside the microchannel. Only the capture electrode is in the microchannel, and thus, the electric field gradient toward the capture electrode is increased in the microchannel. This is achieved by an electric field applied through an insulator via the use of a high-frequency electric field in DEP (Figure 1b). Therefore, bacteria flowing along the flow path are collected by the capture electrode via DEP and flow downstream, resulting in the bacteria flowing in a single line in the vicinity of the downstream outlet. Since the channel is used only for flowing a sample, clogging by bacteria can be prevented by using a channel with a sufficiently large cross-sectional area yet compatible with the range of the dielectrophoretic force.

The dielectrophoretic force is an electrostatic force acting on a particle and arises from the dipole moment induced in the particle by an applied external heterogeneous electric field [30,31].

Equation (1) represents the dielectrophoretic force *F_DEP_* acting on particles of radius *r_p_*:

(1) $$F_{DEP}=2\pi r_{p}^{3}\varepsilon_{m}Re\left[K\left(\omega \right)\right]\nabla |E^{2}$$

where *ε_m_* is the dielectric constant of the solvent, ∇ is the vector derivative operator, and *Re*[*K*(*ω*)] is the real part of the Clazius–Mossotti equation represented by Equation (2):

(2) $$K\left(\omega \right)=\frac{\varepsilon_{p}^{*}-\varepsilon_{m}^{*}}{\varepsilon_{p}^{*}+2\varepsilon_{m}^{*}}$$

where ε_p_* and ε_m_* are the complex dielectric constants of particles and solutions and are represented by Equation (3):

(3) $$\varepsilon^{*}=\varepsilon -\frac{2\sigma }{\omega }j=\varepsilon -\frac{\sigma }{\pi f}j$$

where *ε* is the dielectric constant, *σ* is the conductivity, *ω* is the angular frequency, and *f* is the frequency of the applied alternating current.

From Equations (2) and (3), since *Re* [*K*(*ω*)] depends on frequency, the force exerted on the particle also varies with frequency. If *Re*[*K*(*ω*)] is positive, a positive dielectrophoretic force is applied and the particles are forced in the direction of increasing electric field strength. In the proposed device, as shown in Figure 1b, the electric field is concentrated on the capture electrode, and the positive dielectrophoretic force is utilized to collect bacteria in the flow path onto the capture electrode on which the electric field is concentrated.

### 2.2. Numerical Analysis

**Figure 2:**
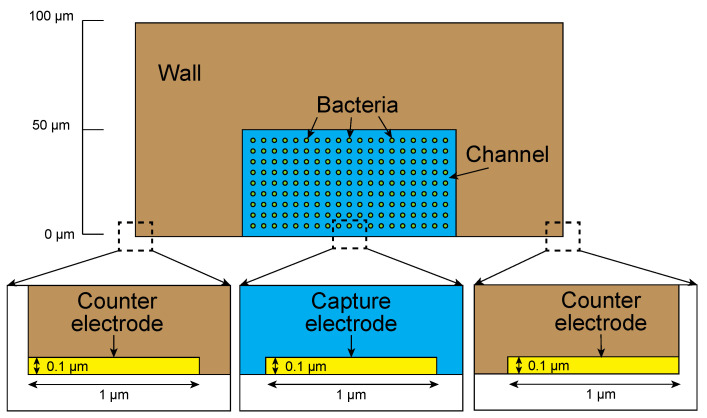
Analysis space in the numerical analysis.

We designed the shapes and arrangements of the electrodes and microchannel by analyzing the movement of particles by the dielectrophoretic force in the microchannel using the commercially available finite element method analysis software package COMSOL (COMSOL 5.6, COMSOL Inc., Stockholm, Sweden). The applied electric potential is assumed to remain constant along the capture and the counter electrodes. Therefore, the numerical calculation can be reduced to a two-dimensional problem of any cross section. The calculation was conducted to calculate the trapping behavior by considering only the dielectrophoretic force and the Stokes drag force in the absence of fluid flow. A model of the analysis space is shown in Figure 2, and the physical properties are shown in Table 1. The conductivity (*σ_m_*) and dielectric constant (*ε_m_*) values are those of water. The conductivity (*σ_p_*) and dielectric constant (*ε_p_*) values of the particles were based on the physical properties of *Escherichia coli* [32], and the external forces acting on the particles are the dielectrophoretic force given by Equation (1) and the Stokes drag force F*_D_* given by Equation (4).

(4) $$F_{D}=\frac{18\mu }{\rho_{p}d_{p}^{2}}m_{p}\left(u-v\right)$$

where *μ* is the kinematic viscosity, *m_p_* is the mass of a bacterium, **v** is the velocity of a bacterium, **u** is the velocity of the liquid, *ρ_p_* is the density of a bacterium, and *d_p_* is the diameter of a bacterium.

The physical properties of the channel structure were based on those of polydimethylsiloxane (PDMS), a silicone rubber [33,34]. The thickness of the electrode was 100 nm, and an AC voltage was applied. As an initial condition, the bacteria were placed in the 9 × 19 lattice pattern at 5 μm intervals in the channel without fluid flow. Under the above boundary, physical property, and initial conditions, we determined the electrode width dependence of the capture state at the capture electrode.

The numerical analysis in COMSOL for the electric field and dielectrophoresis were carried out in electric current interface using Equations (5) to (7).

(5) ∇·J=Q

(6) $$J=\sigma E+j\omega D+J_{e}$$

(7) E=−∇V 

where σ, ω, D, V, J, and Q are conductivity, angular frequency, electric displacement, voltage, external current density, and charge density, respectively. Electrical insulating boundary conditions were set at all boundaries, except for the electrodes. All three electrodes are modeled as perfect conductors. The boundary condition of the capture electrodes was set as the voltage terminal with a voltage of ± 100 V_pp_, while 0 V was applied to the two counter electrodes. Particle tracing interface was coupled with the electric current interface and the laminar flow interface to predict the external forces F*_D_* exerted on the particles. Particle tracing interface calculated the particle momentum and trajectory by solving the motion given by Equation (8).

(8) $$\frac{d\left(m_{p}v\right)}{\mathrm{dt}}=F_{t}$$

The freezing wall boundary conditions were set at all boundary walls inside the channel.

The model of the analysis space was meshed using an extremely fine free triangular mesh having maximum and minimum element sizes of 2 µm and 0.004 μm, respectively, for the calculation domain, with a total of 32,743 mesh elements. The electric current interface was initially solved in the frequency domain. The applied electric potential V was approximated by a polynomial of low order at each mesh point in the calculation. The values of the parameters describing the dielectrophoretic behavior of the particles, such as the electrical field *E* and the gradient *E*^2^, can be obtained from the numerical solution. We then calculated the particle tracing interface in time-dependent mode to evaluate the distribution of the electric field to predict the DEP force and Stokes drag force acting on the particles to obtain the trajectory of the particles inside the microchannel.

### 2.3. Fabrication

The device was fabricated by conventional photolithography and micro-molding.

This device contains a channel chip and an electrode chip bonded together. The fabrication method is conventional and thus described briefly. The channel-chip was fabricated from a PDMS silicone elastomer (KE-106, Shin-Etsu Chemical, Tokyo, Japan). First, a negative resist of SU-8 (SU-8 3050, KAYAKU Advanced Materials, Tokyo, Japan) was spin-coated on a 4-inch silicon wafer at 3000 rpm for 30 s. It was then pre-baked on a hot plate at 95 °C for 15 min and exposed through the photomask of the microchannel using UV light at a wavelength of 365 nm at 240 mJ. The channel was subsequently heated on a hot plate at 95 °C for 5 min and developed (SU-8 developer, KAYAKU Advanced Materials, Tokyo, Japan) to form the negative structure of the microchannel. A release agent (DURASURF831-TH, Daikin, Oosaka, Japan) was coated on the surface of the mold by dipping for several seconds. KE-106 was mixed with the polymerization agent at 10:1 volume ratio, poured onto the mold, degassed in a desiccator, and then heat-cured at 120 °C for 2 h using an oven. The dimensions of the fabricated channel chip were channel width 100 μm, channel height 50 μm, and channel length 20 mm.

**Figure 3:**
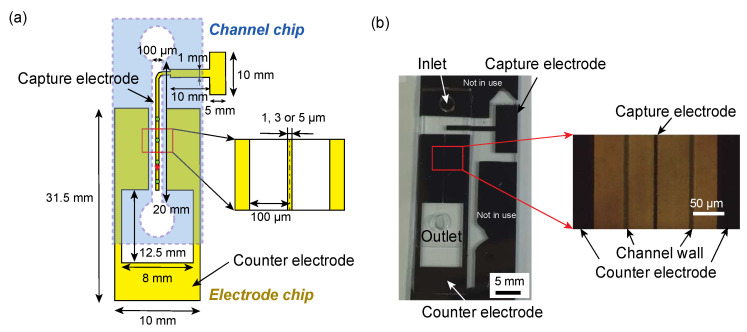
Single-bacteria arraying device: (**a**) an illustration of the device, and (**b**) a photograph of the device and an enlarged view around the capture electrode.

The electrode chip was fabricated by patterning Cr (about 100 nm thick) deposited on a glass substrate using conventional photolithography. First, the photoresist AZP-1350 was spin-coated on the vacuum-evaporated Cr surface on a quartz glass slide at 3000 rpm for 30 s. The photomask in the shape of the electrode was transferred to the photoresist by exposing 365 nm UV light, 60 mJ, through the photomask. Next, the photoresist was developed with a developer (NMD-3, TOKYO OHKA KOGYO, CO., LTD., Kanagawa, Japan) and Cr was patterned to the electrode shape by etching with a Cr etchant. A fluorine-based coating agent (SFE-DP02H, AGCs) was spin-coated at 2000 rpm for 30 s on the fabricated electrode to prevent nonspecific adhesion of bacteria. Finally, the fabricated channel chip was aligned and hermetically bonded to the electrode chip and used as the device. An overall photograph of the fabricated device and an enlarged view around the capture electrode are shown in Figure 3b.

### 2.4. Materials

K-12 strain of *Escherichia coli* (20135, RIKEN BRC) and the lactic acid bacterium *Pediococcus pentosaceus* (AOK-L4140, Akita Konno Co., Ltd., Akita, Japan) were used as bacterial samples. LB medium (12780-052, Thermo Fisher, MA, USA) was used to cultivate *E. coli*, which was cultured in an incubator at 37 °C for 4 h. MRS medium (288130, Becton Dickinson, NJ, USA) was used for *P*. *pentosaceus,* which was cultured at 25 °C for 24 h. After incubation, a 1 mL portion of culture was centrifuged at 5000 *g* for 2 min, the pellet was suspended in pure water, and then, the cells were stained with SYBR Gold (S11494, Thermo Fisher) at a final concentration of 0.5%. Finally, the surfactant Triton-X (9002-93-1, Sigma-Aldrich, MI, USA) was added to the sample at a final concentration of 0.05% to prevent bacterial adhesion onto the inner surface of the microchannel.

### 2.5. Experimental Setup

The flow rate of bacteria in the channel was controlled by applying negative pressure to the outlet using a micropump (RP-HX01S-1A-DC3VS, Aquatech, Oosaka, Japan), and then, the behavior of the bacteria in the microchannel was observed when the dielectrophoretic force was applied. The electrical parameters were set based on preliminary experiments and the simulation results. The voltage used was within the range where neither electrolysis by the electrode occurred nor heat was generated. The frequency chosen was 1 MHz, as this frequency generates a sufficiently strong positive dielectrophoretic force within the bandwidth of the amplifier. The voltage was generated (waveform of 1 MHz, 10 V_pp_) using a function generator (SG4322, IWATSU ELECTRIC CO., LTD., Tokyo Japan) and amplified to 200 V_pp_ using an amplifier (BA4825, NF CORPORATION, Tokyo Japan). Bacterial movement was observed under a fluorescence microscope (LG-PS2, Olympus Corporation, Tokyo, Japan) to determine how bacteria are transported to the electrode by DEP while flowing in the microchannel. The whole view of the setup is shown in Supplementary Figure S1.

## 3. Results

### 3.1. Numerical Analysis of the Motion of Bacteria

**Figure 4:**
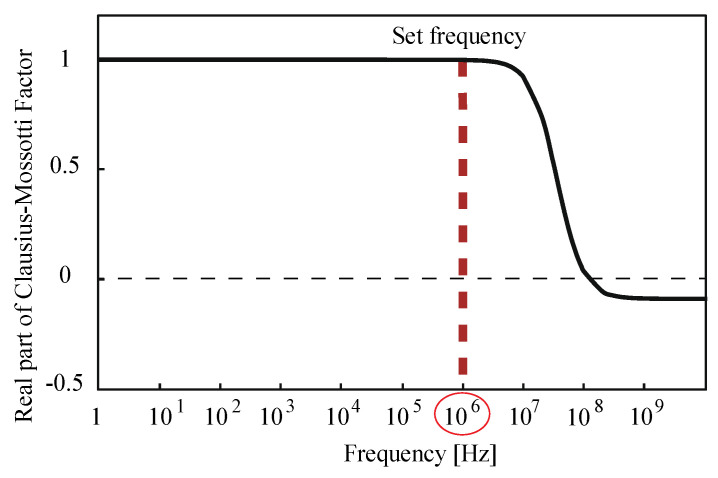
Calculated values of the Clasius-Mossotti equation using the parameters in Table 1: the frequency of 1 MHz was determined as high as possible in the range of positive dielectrophoresis to suppress electrolysis of the electrode from this.

The driving voltage and frequency of the DEP force used in the numerical analysis and experiments were obtained based on the preliminary experiments and calculation results. The applied voltage 200 V_pp_ used was the maximum voltage within the range where neither electrolysis by the electrode occurred nor heat was apparently generated in the preliminary experiments. The frequency 1 MHz was determined as high as possible in the range of positive dielectrophoresis to suppress electrolysis of the electrode from the numerical calculation, as shown in Figure 4. We also confirmed that 1 MHz was the best in the preliminary experiment and used it for the numerical analysis and experiment.

**Figure 5:**
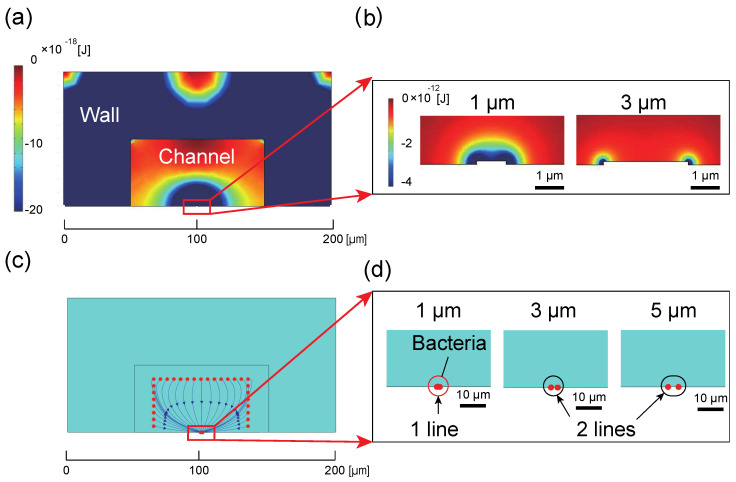
Analysis results: (**a**) the potential energy of the dielectrophoretic force in the cross section of the microchannel, (**b**) an enlarged view of the vicinity of the capture electrode, (**c**) the trajectory of particle tracing, and (**d**) a view of the capture results for each electrode width. Poor contrast between the particles, background, and electrode made it difficult to distinguish these components, and thus, the background was dyed light blue.

The results of the finite element analysis are shown in Figure 5, which represents the potential energy of the dielectrophoretic force (U_D_), represented by Equation (9).

(9) $$U_{D}=-\int \;F_{DEP}\cdot dr=\;-2\pi r_{p}^{3}\varepsilon_{m}Re\left[K\left(\omega \right)\right]\left|E^{2}\right|$$

**Figure 6:**
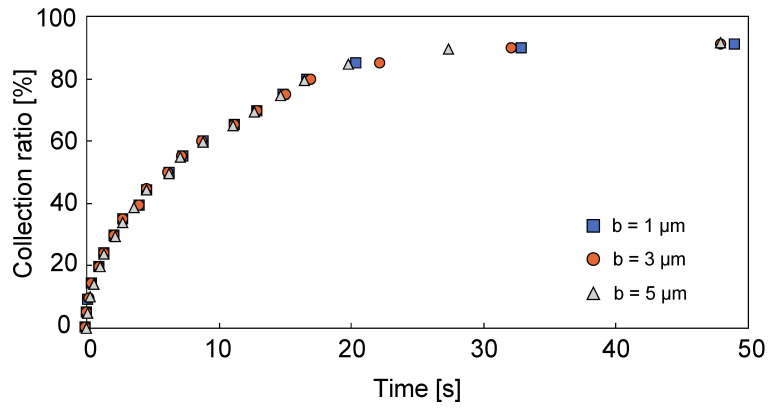
Time evolution of the bacterial capture rate by numerical simulation: the time trend of the capture rate remains essentially unchanged regardless of the electrode width.

Bacteria in the channel are eventually trapped on the electrode because they move to the point of lowest potential. Figure 5b shows the potential energy of the DEP force in the vicinity of the electrode, where the electric field is strongest at the edge of the electrode, as shown in Figure 5c. Therefore, if the capture electrode is wide, bacteria are arranged in two rows on both edges of the electrode, as shown in Figure 5d. On the other hand, if the electrode is narrow, the spacing between the edges decreases and the bacteria are arranged in one row on the electrode. We also calculated the cross-sectional size of the microchannel where DEP acts effectively. As is apparent from Figure 5a, since the local minimum potentials are formed at the center, the left, and the right corners of the channel ceiling, the bacteria in these vicinities cannot be collected by the capture electrode because the bacteria are attracted toward the ceiling. This makes it difficult to collect bacteria in 100% of the channel cross-sectional area: about 80% of the area was available for collecting bacteria experimentally. Figure 6 shows the collection ratio of bacteria collected when initially arranged 171 bacteria in the 9 × 19 lattice pattern at 5 μm intervals in the microchannel for each width of capture electrode. The results indicate that the trend in the number of captured bacteria remains essentially unchanged regardless of the width of the capture electrode, with more than 80% of the bacteria in the channel collected in about 10 s for *P. pentosaceus* and about 16 s for *E. coli* when using a capture electrode with a cross section 100 µm wide and 50 µm high.

### 3.2. One-Dimensional Arrangement of Bacteria

**Figure 7:**
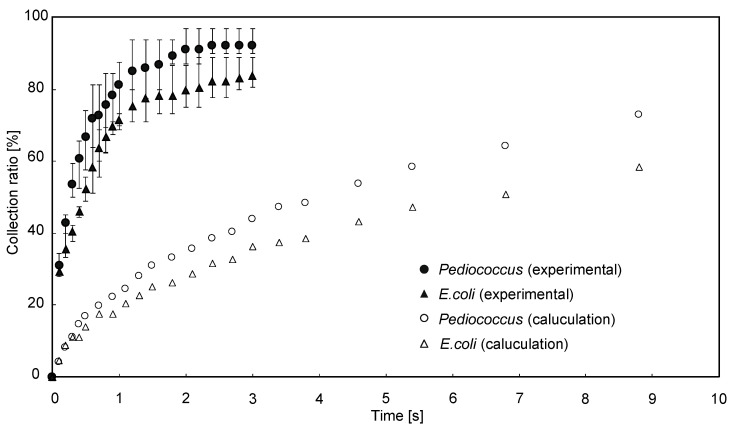
Time variation in the number of captured bacteria: measurements were performed at least three times to confirm reproducibility. The trapping amount saturates at a value of more than 80% in about 2 s.

We measured the time evolution of the number of bacteria trapped on the capture electrode using an external dielectrophoretic force under no-flow conditions to evaluate capture efficiency. The number of bacteria collected over time is shown in Figure 7. Numerical analysis was calculated by the equivalent diameter of lactic acid bacteria (1.3 μm for lactic acid bacteria and 1 μm for *E. coli*) [35]. The times required to capture 80% of the bacterium were about 10 s for *P. pentosaceus* and about 16 s for *E. coli* in the numerical analysis, whereas the required times were about 1 s for *P. pentosaceus* and about 2 s for *E. coli* in the experiments. In comparison to the experimental and calculated values in Figure 7, the trend in time response of the capture rate is similar for both the numerical analysis and the experiments. However, the time required to capture 80% of the bacteria experimentally was more than 8 times faster than the simulation. Despite the differences between the numerical analysis and actual experiments, we nonetheless demonstrated the underlying concept.

**Figure 8:**
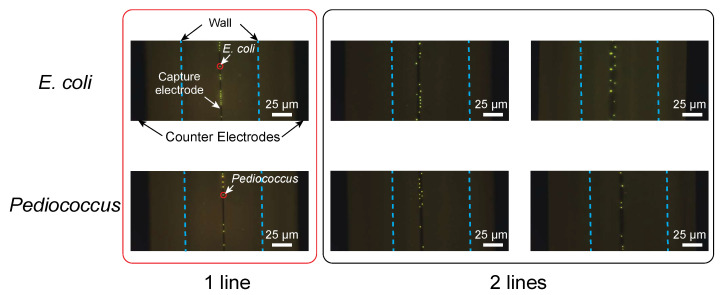
Electrode width dependence of the captured bacteria: Both *E. coli* at a flow rate of 590 µm/s and *P. pentosaceus* at a flow rate of 590 µm/s are similarly arranged in one row when the electrode was about 1 μm or less in width and are arranged in two rows at both edges of the electrode when the electrode was thick (3 μm to 5 μm).

Figure 8 shows how bacteria collected on the electrode as the width of the capture electrode varied. Figure 8 was obtained at the flow rate of 590 µm/s for *E. coli* and 560 µm/s for *P. pentosaceus*. Both the Gram-negative bacterium *E. coli* and the Gram-positive bacterium *P. pentosaceus* were similarly arranged in two rows at the edges of both side of the electrode when the electrode was thick (3 μm to 5 μm). On the other hand, when the electrode width was 1 μm or less, the bacteria were arranged in one row on the electrode, as shown in the figure. Consequently, bacteria can be ordered in a one-dimensional arrangement if the electrode is approximately 1 μm wide, which is equal to or less than the size of a bacterium.

### 3.3. Off-Axis Manipulation of Bacteria

**Figure 9:**
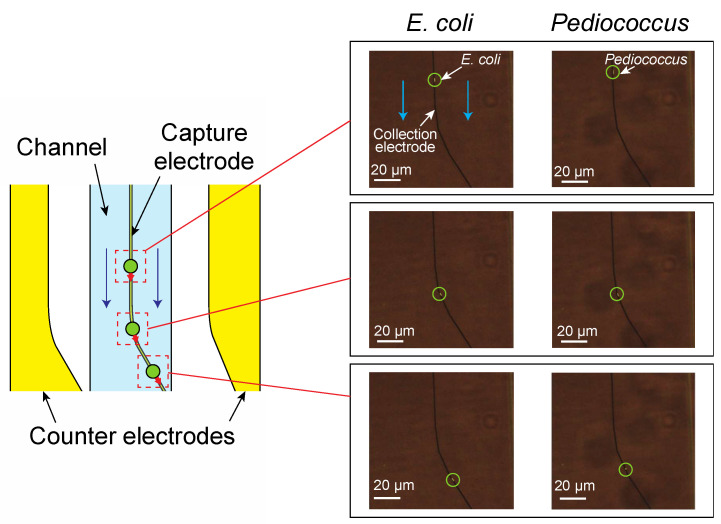
Off-axis manipulation of the bacteria: the bacteria move along the electrode in a direction oblique to the flow.

Some applications, such as cell sorting, require bacteria moving against liquid flow for cell fractionation. We thus evaluated the device with bacteria moving in the off-axis direction by flowing bacteria while trapping them on an electrode bent to the right at 30° to the flow direction. The results verified that the bacteria could move in directions other than the flow direction along the edge of the electrode. Figure 9 shows the off-axis manipulation of *E. coli* and *P. pentosaceus*. The bacteria could flow either parallel to the flow or 30° oblique on the electrode. Although the relationship between flow angle, flow velocity, and applied voltage is currently unclear, bacterial movement can clearly be controlled in off-axis directions.

## 4. Discussion

Experimentally, we succeeded in collecting bacteria much faster than suggested by numerical analysis. Although it is desirable to collect bacteria faster, it is problematic that the prediction of the collection rate by numerical calculation was unsuccessful. Experimental results may include the effects of electric double layer (EDL) and electrohydrodynamic (EHD) convection [36,37]. Microparticles in fluids, such as cells, bacteria, and charged non-bioparticles, are surrounded by an EDL. Counterion relaxation and conduction in an EDL affect the behavior of particles in a dielectrophoretic force significantly. As the particle size decreases, the EDL thickness becomes larger relative to the particle size, especially when the conductivity of the solvent is low, such as the low concentration of electrolyte used in positive DEP in this work. Numerical analysis of the dielectrophoretic collection did not take into account the effects of EDL and EHD convection, thereby leading to a large difference in the calculated and experimentally determined collection rates. The experiments were carried out at voltages and frequencies where no apparent convection occurred, but there could have been weak EHD convection undetectable by microscopic observation. Moderate EHD convection is known to increase the collection efficiency of submicron particles by dielectrophoretic force [38], further suggesting that EHD convection could have helped increase the collection efficiency. Evaluating the effect of EHD convection is a future challenge since it could not be observed microscopically, even though it apparently improved collection efficiency.

Detailed experimental characterization of the device will be reported in the future. The present results nonetheless demonstrate that bacteria could flow either parallel to the liquid flow or 30° oblique on the electrode, suggesting that the method can also be used for separation procedures, e.g., sorting the outlets to collect them depending on the type of bacteria.

Although dielectrophoretic forces have the disadvantage that the force exerted decreases as the particle size decreases because they are volumetric forces, they are also known to act effectively on nanometer-scale particles such as proteins. Therefore, it may be possible to move viral and exosome bionanoparticles arranged in a single line using a smaller capture electrode. Because such bionanoparticles are difficult to manipulate with existing flow cytometers, this approach would not only miniaturize conventional methodologies but also would find value as a platform technology for bionanoparticle manipulation.

## 5. Conclusions

Here, we proposed and demonstrated a microfluidic device for achieving the manipulation of one-dimensional arrays of bacteria using the dielectrophoretic force generated on an electrode rail. We first showed the feasibility of our method by numerical analysis using the finite element method. Based on the various parameters obtained from the numerical analysis, we experimentally evaluated the device and found that, when the electrode width was narrower than the diameter of a bacterium, as indicated by the numerical analysis, the solution can be delivered downstream while arranging the bacteria in one row on the capture electrode. The capture efficiency of the one-dimensional array was 80% or more within 2 s. In addition, since some cell-sorting applications require bacteria to move against the liquid flow, such as during fractionation, we demonstrated that bacteria can move in the axial direction tilted 30° from the flow direction. In the future, we will develop a device for evaluating bacteria with single-cell accuracy by incorporating a sensor downstream of this device and by applying it to bacterial sorters and related devices.

## Figures and Tables

**Table 1:** Physical properties for numerical analysis.

Parameter	Value
$r_{p}$	0.5 (µm)
$\varepsilon_{p}$	60
$\varepsilon_{m}$	80
$\sigma_{p}$	0.44 (S/m)
$\sigma_{m}$	5.56 (µS/m)
μ	1 (mPa)
$\rho_{p}$	1000 (kg/$m^{3}$)
$d_{p}$	1 (µm)

## Supplementary Materials

The following are available online at https://www.mdpi.com/2072-666X/12/2/123/s1, Figure S1: Experimental setup.

## References

[1] Lazcka O., Campo F.J.D., Munoz F.X. (2007). Pathogen detection: A perspective of traditional methods and biosensors. Biosens. Bioelectron..

[2] Leonard P., Hearty S., Brennan J., Dunne L., Quinn J., Chakraborty T., O’Kennedy R. (2003). Advances in biosensors for detection of pathogens in food and water. Enzym. Microb. Technol..

[3] Foudeh A.M., Fatanat Didar T., Veres T., Tabrizian M. (2012). Microfluidic designs and techniques using lab-on-a-chip devices for pathogen detection for point-of-care diagnostics. Lab Chip.

[4] Law J.W.-F., Ab Mutalib N.-S., Chan K.-G., Lee L.-H. (2015). Rapid methods for the detection of foodborne bacterial pathogens: Principles, applications, advantages and limitations. Front. Microbiol..

[5] Piyasena M.E., Graves S.W. (2014). The intersection of flow cytometry with microfluidics and microfabrication. Lab Chip.

[6] Bene M.C. (2017). Microfluidics in flow cytometry and related techniques. Int. J. Lab. Hematol..

[7] Bernabini C., Holmes D., Morgan H. (2011). Micro-impedance cytometry for detection and analysis of micron-sized particles and bacteria. Lab Chip.

[8] Petchakup C., Tay H.M., Li K.H.H., Hou H.W. (2019). Integrated inertial-impedance cytometry for rapid label-free leukocyte isolation and profiling of neutrophil extracellular traps (NETs). Lab Chip.

[9] Apetrei A., Ciuca A., Lee J.-k., Seo C.H., Park Y., Luchian T. (2016). A Protein Nanopore-Based Approach for Bacteria Sensing. Nanoscale Res. Lett..

[10] Walter A., Marz A., Schumacher W., Rosch P., Popp J. (2011). Towards a fast, high specific and reliable discrimination of bacteria on strain level by means of SERS in a microfluidic device. Lab Chip.

[11] Lyu Y., Yuan X., Glidle A., Fu Y., Furusho H., Yang T., Yin H. (2020). Automated Raman based cell sorting with 3D microfluidics. Lab Chip.

[12] Lin G., Makarov D., Schmidt O.G. (2017). Magnetic sensing platform technologies for biomedical applications. Lab Chip.

[13] Chicharo A., Martins M., Barnsley L.C., Taouallah A., Fernandes J., Silva B.F.B., Cardoso S., Dieguez L., Espina B., Freitas P.P. (2018). Enhanced magnetic microcytometer with 3D flow focusing for cell enumeration. Lab Chip.

[14] Song Y., Zhang H., Chon C.H., Chen S., Pan X., Li D. (2010). Counting bacteria on a microfluidic chip. Anal. Chim. Acta.

[15] Lopez P.A., Hulspas R. (2020). Special Issue on Enhancement of Reproducibility and Rigor. Cytom. Part A.

[16] Kuan D.-H., Huang N.-T. (2020). Recent advancements in microfluidics that integrate electrical sensors for whole blood analysis. Anal. Methods.

[17] Huang C.-T., Weng C.-H., Jen C.-P. (2011). Three-dimensional cellular focusing utilizing a combination of insulator-based and metallic dielectrophoresis. Biomicrofluidics.

[18] Zhao S., Wu M., Yang S., Wu Y., Gu Y., Chen C., Ye J., Xie Z., Tian Z., Bachman H. (2020). A disposable acoustofluidic chip for nano/microparticle separation using unidirectional acoustic transducers. Lab Chip.

[19] Witte C., Reboud J., Wilson R., Cooper J.M., Neale S.L. (2014). Microfluidic resonant cavities enable acoustophoresis on a disposable superstrate. Lab Chip.

[20] Zhang J., Yan S., Yuan D., Alici G., Nguyen N.-T., Ebrahimi Warkiani M., Li W. (2016). Fundamentals and applications of inertial microfluidics: A review. Lab Chip.

[21] Zhou Y., Ma Z., Ai Y. (2020). Dynamically tunable elasto-inertial particle focusing and sorting in microfluidics. Lab Chip.

[22] Fiedler S., Shirley S.G., Schnelle T., Fuhr G. (1998). Dielectrophoretic Sorting of Particles and Cells in a Microsystem. Anal. Chem..

[23] Choongho Y., Vykoukal J., Vykoukal D.M., Schwartz J.A., Li S., Gascoyne P.R.C. (2005). A three-dimensional dielectrophoretic particle focusing channel for microcytometry applications. J. Microelectromech. Syst..

[24] Muller T., Gradl G., Howitz S., Shirley S., Schnelle T., Fuhr G. (1999). A 3-D microelectrode system for handling and caging single cells and particles. Biosens. Bioelectron..

[25] Xuan X., Zhu J., Church C. (2010). Particle focusing in microfluidic devices. Microfluid. Nanofluidics.

[26] Chu H., Doh I., Cho Y.-H. (2009). A three-dimensional (3D) particle focusing channel using the positive dielectrophoresis (pDEP) guided by a dielectric structure between two planar electrodes. Lab Chip.

[27] Cheng I.-F., Chang H.-C., Hou D., Chang H.-C. (2007). An integrated dielectrophoretic chip for continuous bioparticle filtering, focusing, sorting, trapping, and detecting. Biomicrofluidics.

[28] Ateya D.A., Erickson J.S., Howell P.B., Hilliard L.R., Golden J.P., Ligler F.S. (2008). The good, the bad, and the tiny: A review of microflow cytometry. Anal. Bioanal. Chem..

[29] Kim M., Jung T., Kim Y., Lee C., Woo K., Seol J.H., Yang S. (2015). A microfluidic device for label-free detection of Escherichia coli in drinking water using positive dielectrophoretic focusing, capturing, and impedance measurement. Biosens. Bioelectron..

[30] Morgan H., Hughes M.P., Green N.G. (1999). Separation of Submicron Bioparticles by Dielectrophoresis. Biophys. J..

[31] Pethig R. (2010). Review Article-Dielectrophoresis: Status of the theory, technology, and applications. Biomicrofluidics.

[32] Holzel R. (1999). Non-invasive determination of bacterial single cell properties by electrorotation. Biochim. Et Biophys. Acta Mol. Cell Res..

[33] National Astronomical Observatory of Japan (2020). Chronological Scientific Tables.

[34] Molberg M., Leterrier Y., Plummer C.J.G., Walder C., Lowe C., Opris D.M., Nuesch F.A., Bauer S., Manson J.-A.E. (2009). Frequency dependent dielectric and mechanical behavior of elastomers for actuator applications. J. Appl. Phys..

[35] Jones R.B. (2008). Electromechanics of Particles.

[36] Washizu M., Suzuki S., Osamu K., Nishizaka T., Shinohara T. (1994). Molecular dielectrophoresis of biopolymers. IEEE Trans. Ind. Appl..

[37] Muller T., Gerardino A., Schnelle T., Shirley S.G., Bordoni F., Gasperis G.D., Leoni R., Fuhr G. (1996). Trapping of micrometre and sub-micrometre particles by high-frequency electric fields and hydrodynamic forces. J. Phys. D Appl. Phys..

[38] Dash S., Mohanty S. (2014). Dielectrophoretic separation of micron and submicron particles: A review. Electrophoresis.

