# Supplementary material for: One-Dimensional Flow of Bacteria on an Electrode Rail by Dielectrophoresis: Toward Single-Cell-Based Analysis
Source: Micromachines (Basel). 2021 Jan 24;12(2):123. doi: 10.3390/mi12020123 (PMC7911595; doi:10.3390/mi12020123)
Supplement: Supplementary file 1 [file micromachines-12-00123-s001.pdf]

Supplementary Materials

One-Dimensional Flow of Bacteria on an Electrode Rail by Dielectrophoresis: Toward Single-Cell-Based Analysis

Yukihiro Yamaguchi and Takatoki Yamamoto*

Department of Mechanical Engineering, School of Engineering, Tokyo Institute of Technology; 2-12-1 Ookayama, Meguro-ku, Tokyo 152-8550, Japan
yamamoto.t.ba@m.titech.ac.jp
*Correspondence: yamamoto.t.a@m.titech.ac.jp; Tel.: +81-3-5734-3182

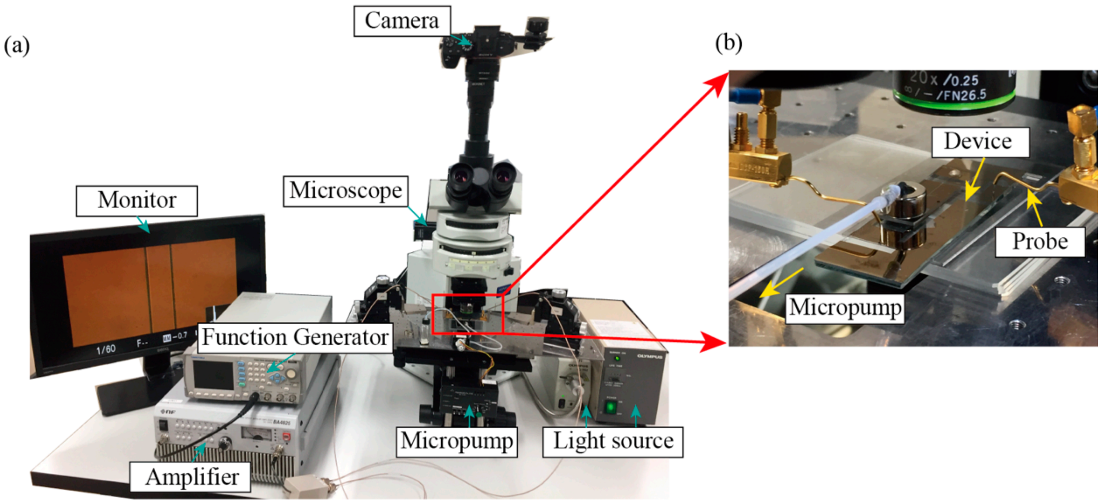

Figure S1. Experimental setup. The voltage was generated (waveform of 1 MHz, 10 Vpp) using a function generator (SG4322, IWATSU ELECTRIC CO., LTD.) and amplified to 200 Vpp using an amplifier (BA4825, NF CORPORATION). Bacterial movement was observed under a fluorescence microscope (LG-PS2, Olympus Corporation) to determine how bacteria are transported to the electrode by DEP while flowing in the microchannel. The flow rate of bacteria in the channel was controlled by applying negative pressure to the outlet using a micropump (RP-HX01S-1A-DC3VS, Aquatech)
